# Lectin binding profiles of SSEA-4 enriched, pluripotent human embryonic stem cell surfaces

**DOI:** 10.1186/1471-213X-5-15

**Published:** 2005-07-21

**Authors:** Alison Venable, Maisam Mitalipova, Ian Lyons, Karen Jones, Soojung Shin, Michael Pierce, Steven Stice

**Affiliations:** 1 Department of Biochemistry and Molecular Biology, University of Georgia, Athens, Georgia, USA; 2 Department of Animal and Dairy Sciences, University of Georgia, Athens, Georgia, USA; 3 BresaGen Inc, Athens, Georgia, USA

## Abstract

**Background:**

Pluripotent human embryonic stem cells (hESCs) have the potential to form every cell type in the body. These cells must be appropriately characterized prior to differentiation studies or when defining characteristics of the pluripotent state. Some developmentally regulated cell surface antigens identified by monoclonal antibodies in a variety of species and stem cell types have proven to be side chains of membrane glycolipids and glycoproteins. Therefore, to examine hESC surfaces for other potential pluripotent markers, we used a panel of 14 lectins, which were chosen based on their specificity for a variety of carbohydrates and carbohydrate linkages, along with stage specific embryonic antigen-4 (SSEA-4), to determine binding quantitation by flow cytometry and binding localization in adherent colonies by immunocytochemistry.

**Results:**

Enriching cells for SSEA-4 expression increased the percentage of SSEA-4 positive cells to 98–99%. Using enriched high SSEA-4-expressing hESCs, we then analyzed the binding percentages of selected lectins and found a large variation in binding percentages ranging from 4% to 99% binding. *Lycopersicon *(tomato)*esculetum *lectin (TL), *Ricinus communis *agglutinin (RCA), and *Concanavalin A *(Con A) bound to SSEA-4 positive regions of hESCs and with similar binding percentages as SSEA-4. In contrast, we found *Dolichos biflorus *agglutinin (DBA) and *Lotus tetragonolobus *lectin (LTL) did not bind to hESCs while *Phaseolus vulgaris *leuco-agglutinin (PHA-L), *Vicia villosa *agglutinin (VVA), *Ulex europaeus *agglutinin (UEA), *Phaseolus vulgaris *erythro-agglutinin (PHA-E), and *Maackia amurensis *agglutinin (MAA) bound partially to hESCs. These binding percentages correlated well with immunocytochemistry results.

**Conclusion:**

Our results provide information about types of carbohydrates and carbohydrate linkages found on pluripotent hESC surfaces. We propose that TL, RCA and Con A may be used as markers that are associated with the pluripotent state of hESCs because binding percentages and binding localization of these lectins are similar to those of SSEA-4. Non-binding lectins, DBA and LTL, may identify differentiated cell types; however, we did not find these lectins to bind to pluripotent SSEA-4 positive hESCs. This work represents a fundamental base to systematically classify pluripotent hESCs, and in future studies these lectins may be used to distinguish differentiated hESC types based on glycan presentation that accompanies differentiation.

## Background

Ever since the isolation of human embryonic stem cells (hESCs) in 1998 [1], the implications for their use in a number of disease therapies have been highly regarded. Additionally, these cells also find value as a model to study basic human development. However, in all aspects of ESC research, hESCs must first be appropriately defined or characterized. One way to characterize hESCs is to utilize the large number of glycoproteins and carbohydrates existing on the cell surface as a way to delineate pluripotent or differentiated cell types.

The most common hESC surface pluripotency markers are the stage specific embryonic antigens -3 and -4 (SSEA-3, -4) and tumor rejection antigens-1-60 and -1-81 (TRA-1-60, -1-81). SSEA-3 and -4 are globoseries cell surface glycoproteins that were first used to delineate embryological changes in the developing mouse embryo [2,3]. Both of these antigens were found to recognize sequential regions of a mouse ganglioside epitope, with SSEA-4 (MC813-70 antibody) recognizing the terminal portion of the sequence and SSEA-3 (MC613) recognizing the internal region of he sequence. Thus, two antibodies were used to define this unique embryonic antigen. In mouse embryonic stem cells (mESCs), SSEA-3 and -4 are expressed on the 2–8 cell and morula stages of preimplantation embryos and are also found on unfertilized oocytes; however, there is a loss of expression in the inner cell mass (ICM) of mESCs [2,3]. Yet in hESCs, there is no expression of SSEA-3 or -4 at the 2–8 cell or morula stage; however, these are expressed on the ICM of human blastocysts and on isolated hESCs [4]. It has been well documented that these cell surface carbohydrates change both with development and differentiation in vitro [5,6], but there may be other undiscovered developmentally regulated cell surface carbohydrates.

The hematopoetic field has numerous cell surface antigens which have been identified that define various bone marrow and blood stem cells, such as CD4, CD8, CD34, CD38, CD44, CD45, c-kit, Mac-1, Muc -18, Lin, Sca-1, and Thy-1 [7-11] and there has been some progress in identifying other hESC surface markers that can be used both for defining the pluripotent state and for cell sorting and cell identification. Cell surface markers such as CD9 and CD24 are presented on pluripotent hESC surfaces, while gene expression analysis indicates such genetic markers as REX1 [12], Cripto/TDGF1 [12], OCT-4 [13], DNMT3B [13], LIN28 [13], Nanog [14], and others that can denote pluripotency in hESCs [13]. Since carbohydrate antigens on hESC surfaces could provide further potential markers defining the pluripotent state, we targeted glycosylation patterns on the cell surface of SSEA-4 enriched hESCs using lectins to bind to these glycans. Lectins, carbohydrate binding proteins that recognize diverse sugar structures, have been extensively used to identify and characterize cell surface glycosylation patterns. For example, lectins have been used to investigate metastatic processes in many types of cancer [15-21] and to identify cell types based on presentation of specific cell surface carbohydrates [22-27]. Carbohydrate analysis using lectins has also led to the delineation of embryologic developmental stages in some species. For example, many developmentally regulated glycans identified as lectin receptors on mESCs are displayed on cell surfaces at the preimplantation and implantation stages of development. These include *Concanavalin *A (Con A) defined by the hapten methylmannoside [28], Peanut agglutinin (PNA) from *Arachis hypogaea *defined by the hapten galactose [29], Wheat Germ Agglutinin (WGA) from *Triticum vulgaris *defined by hapten N-acetylglucosamine [30], *Dolichos biflorus *agglutinin (DBA) defined by the hapten N-acetylgalactosamine [31], and *Ricinus communis *agglutinin (RCA) defined by the hapten galactose or lactose [32]. These results suggest that glycans may be involved in cell-cell interactions serving a specific developmental function and also indicate that they can be used as markers to define these stages of mouse embryogenesis.

**Figure 1:**
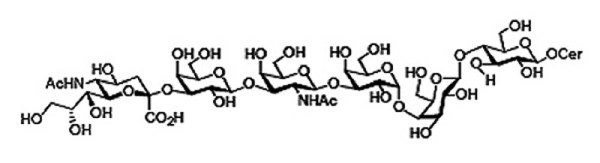
**Structure of Stage Specific Embryonic Antigen 4 (SSEA4). **SSEA-4, one of the most commonly used hESC surface pluripotency markers, is a globoseries glycolipid that is characteristically downregulated upon hESC differentiation.

In this study, we analyzed the pluripotent state of two NIH approved hESC lines, BG01 and BG02. These cell lines have previously been characterized for positive staining of the pluripotency markers TRA-1-60 and -1-81, OCT-4, alkaline phosphatase, and SSEA-3 and -4 [33]. Lectin binding percentages on SSEA-4 enriched hESC surfaces were determined by flow cytometry using a panel of 14 lectins and SSEA-4, which enabled us to probe the surface of pluripotent hESCs for a variety of carbohydrates and carbohydrate linkages. Rosler et al showed that in long-term culture, hESCs maintained pluripotency as determined by measurement of a variety of characteristic markers, including SSEA-4, TRA-1-60 and TRA-1-81 [34]. Moreover, there was a high correlation between expression of SSEA-4 and TRA-1-60 and -1-81, and the presence of these markers correlated with undifferentiated morphology. Thus, we defined pluripotent hESCs as those populations with 98–99% expression of SSEA-4, a developmentally regulated cell surface pluripotency marker that is frequently used to delineate pluripotent hESCs [1,4,5,35]. We were able to obtain this high level of SSEA-4 expression by using magnetic bead sorting to select SSEA-4 expressing cells. The SSEA-4 antibody (MC 813-70) specifically recognizes a carbohydrate chain (Figure 1); thus, we analyzed a panel of lectins that are likely to be presented on hESC surfaces in order to identify other potential markers for pluripotent hESCs. Immunocytochemistry using SSEA-4 and each of the 14 lectins was analyzed to determine localization of lectin binding within adherent hESC colonies and to validate binding specificity of each lectin using appropriate competitive sugars.

We found that a variety of lectins binding unique carbohydrate moieties had distinct binding patterns. Our findings demonstrate that there are many surface carbohydrate antigens that could be exploited to further characterize the pluripotent state of hESCs, and these lectins may provide a source of unique markers with which to characterize subpopulations that exist in colonies of adherent hESCs.

## Results

### Carbohydrate analysis using flow cytometry

**Figure 2:**
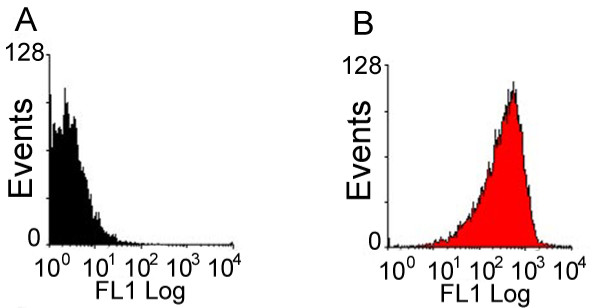
**SSEA-4 expression in enriched hESCs determined by flow cytometry. **The histogram plots of unstained, control hESCs (2A) and cells stained with SSEA-4 antibody after enrichment using magnetic bead sorting at day 0 (2B).

**Figure 3:**
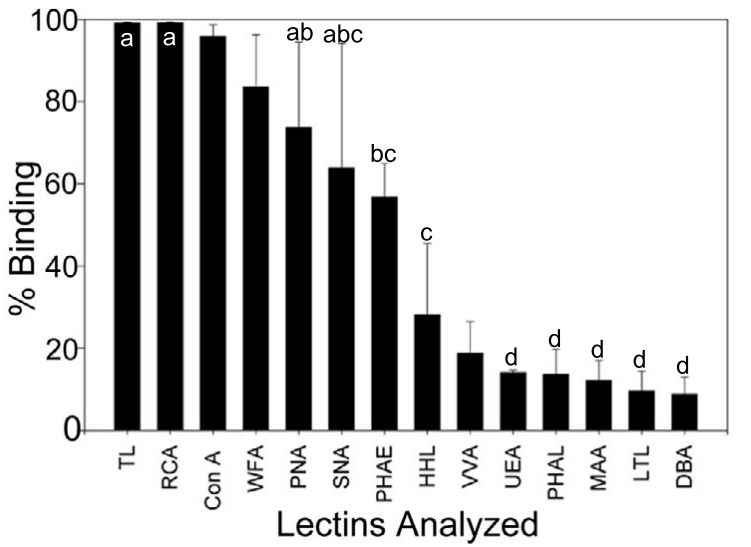
**Quantitation of lectin binding on pluripotent hESC surfaces with 14 different lectins. **The percent of cells with specific carbohydrate expression as determined by flow cytometry using 14 different lectins. The data are means +/- SD of 3 independent assays of BG01 and BG02 hESC lines. hESCs from each line were stained with one of 14 lectins and SSEA-4 immediately following enrichment for SSEA-4 expression. abcd: Means with different letters are significantly different, p < 0.05.

**Figure 4:**
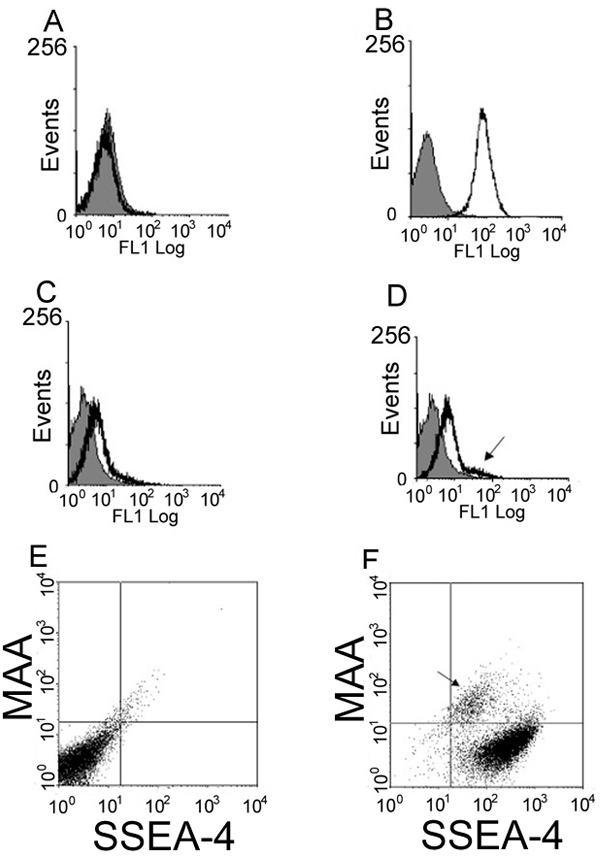
**Flow cytometry histograms of lectin binding in unstained and stained HESCs. **A-D shows histograms of SSEA-4 binding and representative lectins that were used in this study. To validate that double staining can be performed without signal interference, we determined that SSEA-4 expression was not found in the FL1 channel. Figure 4A shows a histogram plot with the overlay image of SSEA4 (black tracing) matching the histogram plot of unstained cells (grey fill). Figure 4B shows a positive peak shift in the histogram overlay with Tomato lectin (TL) in black tracing and unstained cells (grey fill). Figure 4C shows lack of *Lotus tetragonolobus *lectin (LTL) (black tracing) binding in the histogram overlay with unstained cells (grey fill). Figure 4D shows a histogram overlay with MAA (black tracing) binding and shows two peaks -one representing a large population that overlays unstained cells and a smaller population denoted by arrow that shows a smaller population of MAA+/SSEA4+ cells. Figure 4E shows plots of unstained hESCs, and Figure 4F shows subpopulations of cells characterized as SSEA-4+/MAA- or as SSEA-4+/MAA+ cells (arrow).

HESCs were trypsin passaged and selected for SSEA-4. It is important to note that manual passaging would not be feasible for flow cytometry studies, since atleast 250,000 single cells are needed per treatment for flow cytometry analysis. After SSEA-4 enrichment using magnetic bead sorting, 99.3 ± 0.31% of hESCs expressed SSEA-4 (Figure 2). To determine the presence and the percent binding of the 14 chosen lectins on pluripotent hESC surfaces, both karyotypically normal hESC lines (BG01 and BG02) were analyzed using flow cytometry with the panel of lectins and the pluripotency marker, SSEA-4. A broad range of binding percentages was observed (Figure 3). The highest binding percentages were detected using TL, RCA, ConA, WFA, and SNA. Binding percentages for TL, RCA, and ConA were similar to that of SSEA-4, ranging from 98–99% of enriched hESCs. Figure 4B shows a shifted histogram plot of TL as a representative of these high percentage binding lectins. WFA, PNA and SNA were also found to bind to over 60% of hESCs.

Two lectins, DBA and LTL, did not bind to hESCs. Analysis of DBA and LTL histograms showed no peak shifts using these lectins (Figure 4C), and analysis of flow cytometry plots also did not reveal any peak shifts (supplementary data).

Some lectins were found to partially bind to hESC colonies. These lectins revealed two distinct subpopulations of cells that included a SSEA4+/lectin- population and a SSEA4+/lectin+ population. Figure 4D shows a representative histogram plot of MAA which shows a large population of cells that overlap the unstained control cells; however, the arrow indicates another smaller population of cells that show a peak shift representing positive MAA binding. Figure 4E shows the comparison of unstained control cells to the shifted plot of MAA (Figure 4F) in which there are two distinct populations of cells present: a SSEA-4+/lectin- population containing the majority of cells, and a smaller but still distinct SSEA-4+/lectin+ population indicated by the arrow. Lectins that bound in this way included PHA-E, VVA, UEA, PHA-L, and MAA, and these exhibited a large binding range varying from 58% to 13% binding of enriched SSEA-4 positive cells (Figure 3).

### Carbohydrate analysis using immunocytochemistry

**Figure 5:**
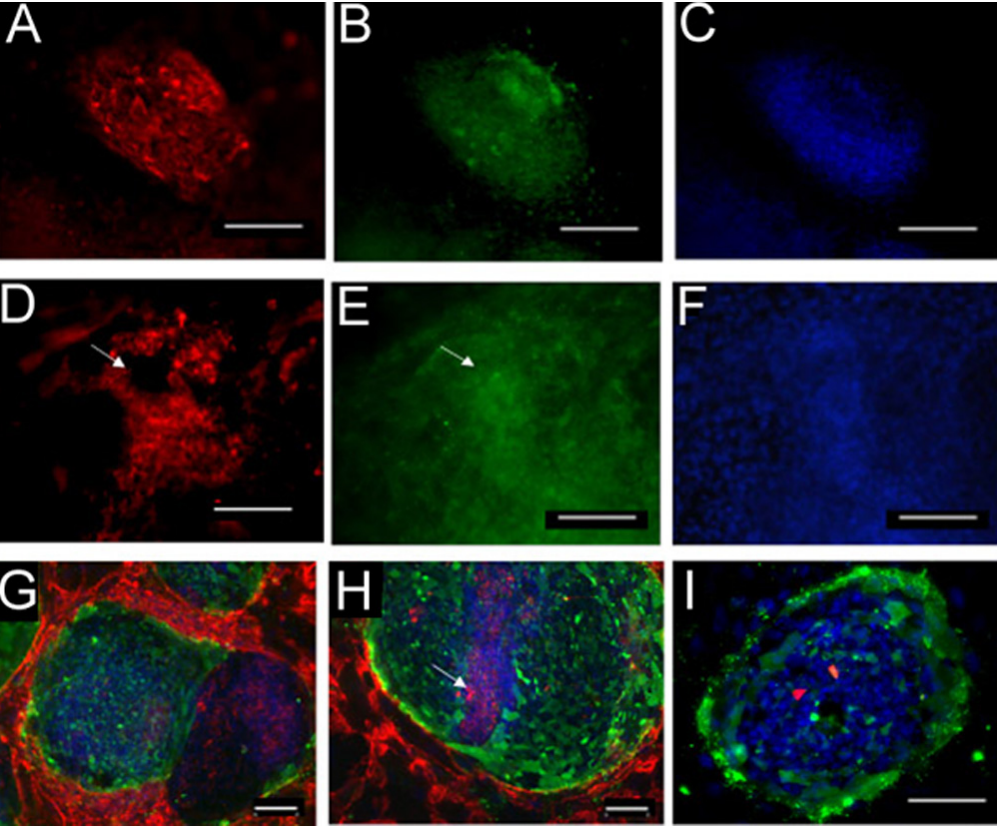
**Carbohydrate expression as determined by lectin binding using immunocytochemistry. **Figure 5A–C shows a hESC colony that represents uniform lectin binding. *Ricinus Communis *agglutinin (RCA) binding in red (5A) is shown throughout this SSEA-4 positive colony in green (5B). The DAPI nuclear stain image (blue) is also shown (5C). Other lectins showed partial binding patterns, such as *Vicia Villosa *agglutinin (VVA) binding (red), which is shown in a hESC colony (5D) that has uniform SSEA-4 antibody binding (green) (5E). Arrows denote distinct SSEA-4 positive regions lacking VVA binding. DAPI nuclear staining (blue) is also shown (5F). PHA-E binding is shown in two separate images in Figure 5G–H. In 5G there are two adjacent colonies, one that expresses strong binding of SSEA-4 antibody (green) and weak to no binding of PHA-E (red), and an adjacent colony showing binding of PHA-E without SSEA-4 antibody binding. (DAPI nuclear staining in blue). 5H, shows another colony with a streak of stacked cells (as determined by high DAPI expression, see arrow) in the middle of the colony that are beginning to lose SSEA-4 expression (green), but have strong PHA-E binding (red). However, the rest of the colony adjacent to this streak of cells is uniformly positive for SSEA-4 but is lacking PHA-E binding. 5I shows lack of DBA binding and presence of SSEA-4 and DAPI staining. Images and scale bars: 5A-G) 20× magnification, 100 μm. H-I) 10× magnification, 100 μm.

Both hESC lines, BG01 and BG02, were analyzed by immunocytochemistry to determine localization of carbohydrates and to determine whether particular staining patterns were present in adherent colonies maintained in culture. Cells were either passaged manually or by trypsinization and we did not observe obvious differences in hESC colony staining patterns between these passage methods. Immunocytochemistry results of all 14 lectins supported our flow cytometry analysis. Five lectins (TL, RCA, ConA, WFA, SNA, and HHL) bound throughout the colonies without any localized patterns of binding. TL, RCA, ConA, and WFA appeared to bind to cells that also expressed SSEA-4. Figure 5A–C shows a hESC colony that represents uniform lectin binding. RCA binding (Figure 5A) is shown throughout a colony that is SSEA-4 positive (Figure 5B) and is also stained with DAPI nuclear stain (Figure 5C).

We also observed interesting patterns of carbohydrate expression for the lectins that showed two subpopulations of cells when analyzed using flow cytomtery. VVA bound to distinct regions of a SSEA-4 positive colony (Figure 5D–F), in contrast to the more uniform binding described above. In Figure 5D the arrow shows a region where there is no VVA binding, but this region is SSEA-4 positive as shown in Figure 5E. The DAPI nuclear stain of this same colony is shown in Figure 5F. Figures 5G & H show PHA-E binding in different colonies. In Figure 5G there are two adjacent colonies with one colony showing strong binding of SSEA-4 antibody (green) and little PHA-E binding (red). The adjacent colony shows binding of PHA-E but a lack of SSEA-4 antibody binding. In Figure 5H there is one colony that shows a streak of stacked cells as determined by the high expression of DAPI staining in the middle of the colony (depicted by arrow). These cells are beginning to lose SSEA-4 antibody binding (green) but have strong PHA-E binding (red). The rest of the colony adjacent to this streak of cells is uniformly positive for SSEA-4 but lacks PHA-E binding.

Using immunocytochemistry we also demonstrated that DBA and LTL did not bind to hESC colonies. Figure 5I shows a hESC colony which is SSEA-4 positive (green) and is stained with DAPI nuclear stain (blue) but shows only debris of the DBA lectin (red) with no regions of DBA binding.

### Validation of lectin binding

**Figure 6:**
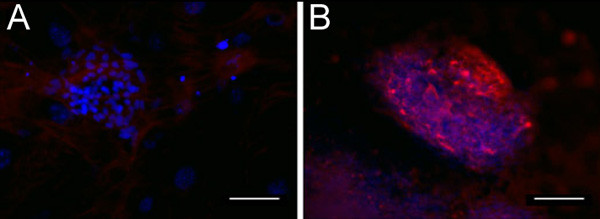
**Addition of competitive sugar inhibitor blocks lectin expression as determined by immunocytochemistry. **Addition of 200 mM galactose can block RCA binding in a hESC colony as shown by lack of RCA binding (6A), but does not affect DAPI stain (blue). However, in the absence of galactose, uniform binding of RCA (red) was detected 6(B). 6A-B) 20× magnification, 100 μm

We used complementary competitive sugars that are known to block lectin binding (listed in Table 1) to validate lectin binding specificity. The concentrations listed in Table 1 were all found to inhibit each lectin's binding as detected by immunocytochemistry. As a representative of competitive sugar inhibition, Figure 6 shows blocking of RCA binding by addition of 200 mM galactose (Figure 6A). In the absence of galactose, RCA binding is evident (red, Figure 6B).

**Table 1:** Comparison of the specificity for monosaccharides and oligosaccharides of a panel of 14 biotinylated lectins used in immunocytochemistry and flow cytometry.

**Lectin Origin**	**Monosaccharide Specificity**	**Inhibitor**
*Concanavalin A *(Con A)	Man or Glc	200 mM α-methylmannoside or α-methyl glucoside
*Phaseolus vulgaris *erythro-agglutinin (PHA-E)	Gal	galactose
*Phaseolus vulgaris *leuco-agglutinin (PHA-L)	Gal	galactose
*Sambucus nigra *agglutinin (SNA)	Sialic Acid	500 mM lactose in acetic acid
*Arachis hypogea *peanut (PNA)	Gal	200 mM galactose
*Vicia villosa *agglutinin (VVA)	GalNAc	200 mM N-acetylgalactosamine
*Maackia amurensis *(MAA)	Gal	200 mM lactose
*Ricinus communis *agglutinin (RCA)	Gal	200 mM galactose or lactose
*Wisteria floribunda *agglutinin (WFA)	GalNAc	200 mM N-acetylgalactosamine
*Ulex europaeus *agglutinin (UEA)	Fuc	50–100 mM L-fucose
*Lotus tetragonolobus *lectin (LTL)	Fuc	50–100 mM L-fucose
*Dolichos biflorus *agglutinin (DBA)	GalNAc	200 mM N-acetylgalactosamine
*Hippeastrum hybrid *lectin (HHL)	Man	100 mM mannose
*Lycopersicon esculetum *tomato (TL)	GlcNAc	Chitin Hydrolysate

## Discussion

This study is the first of which we are aware to use lectins to probe the surface of hESCs in order to analyze carbohydrate presentation. To quantitate lectin binding percentages on pluripotent hESC surfaces using flow cytometry, we first enriched our colonies for SSEA-4 expression. Using enriched pluripotent hESCs, we found a large binding percentage range for this chosen panel of 14 lectins. Some lectins bound throughout the colonies of pluripotent hESCs, and the carbohydrate moieties represented by this group include alpha-linked mannose (Con A), galactosyl(β1–3) N-acetylgalactosamine (PNA), terminal galactose and N-acetylgalactosamine (RCA), GalNAc β4-Gal (WFA), N-acetylglucosamine (TL) and sialic acid alpha 2,6 GalNAc (SNA) and alpha-linked mannose residues only (HHL). These results indicate that there is high expression of these carbohydrates with these particular linkages on pluripotent hESC surfaces. As ConA can bind both mannose and glucose moieties, we see greater lectin binding of ConA as opposed to HHL, which specifically binds only mannose residues. These lectins may be additional markers to denote pluripotent hESCs. Similarly, WFA, PNA and SNA were found to bind to over 60% of hESCs, suggesting that these lectins could be indicative of a pluripotent phenotype as well; however, they do not have binding percentages that correlate with SSEA-4 expression.

We also found that some lectins showed interesting partial binding patterns. Two distinct populations of cells were observed as determined by flow cytometry plot analysis and confirmed by immunocytochemistry using these lectins and SSEA-4 antibody. These hESC populations were identified as either SSEA-4+/lectin+ or SSEA-4+/lectin- regions. Lectins showing this binding pattern included PHA-L, VVA (see Figure 5D–F), UEA, PHA-E (Figure 5G–H), and MAA. Table 1 shows their carbohydrate binding specificities.

As shown by immunocytochemistry of the representative lectin, PHA-E, it appeared that some areas that were beginning to lose expression of SSEA-4, show binding of the lectin (Figure 5G–H). A decrease of SSEA-4 expression can occur even under the most stringent culturing conditions and in this research, is important because it underscores the need for enriching for desired populations of cells prior to experimentation and also supports the hypothesis that cell-cell interactions are important in carbohydrate presentation. As clusters of cells lose SSEA-4 expression, and therefore differentiate, new carbohydrates are presented on the cell surface. Furthermore, these differentiated cells could be indicative of progenitor subpopulations existing in the colonies. For example, another lectin in our study that was found to bind partially to colonies is UEA, which binds fucose moieties and has been determined to be a marker for endothelial cells [16]. The role these partially presented carbohydrates play was not investigated in this study, but others have suggested that certain carbohydrates may serve a developmental function. For example, Panin and colleagues have identified O-fucose on epidermal growth factor-like repeats of Notch, and elongation of O-fucose has been implicated in the modulation of Notch signaling by Fringe [36,37]. Notch receptors and associated proteins are important in a number of signaling pathways that direct cell fate decisions, proliferation and apoptosis.

We also observed that two lectins, DBA and LTL, did not bind hESCs. DBA binds primarily to α-linked N-acetylgalactosamine and LTL binds fucose moieties that are α-linked to GlcNAc. These non-binding lectins suggest that these particular carbohydrates and their associated linkages are either not present on pluripotent hESC surfaces or if they are present, they have been modified and therefore are not susceptible to lectin binding. Interestingly, however, PNA and WFA, which bind to a high percentage of hESCs, bind to β-linked N-acetylgalactosamine. Thus, it appears that the linkage of this carbohydrate is important. Similarly, UEA binds to about 20% of our cells and recognizes fucose moities like LTL. However, the linkages that these lectins recognize are different and cause different binding percent outcomes, further suggesting that the linkage of these carbohydrates are important.

Developmentally regulated cell surface antigens have been identified by monoclonal antibodies in a variety of species and stem cell types. Many of these antigens have proven to be side chains of membrane glycolipids and glycoproteins. For example, the SB10 antigen found on human mesenchymal stem cells was shown to be the activated leukocyte-cell adhesion molecule (ALCAM) that possesses N-linked oligosaccharide side chains [38]. Muramatsu and colleagues discovered a protein called embigin that has a developmentally regulated carbohydrate chain that is lost upon mouse embryogenesis [39]. Furthermore, other researchers showed that the monoclonal antibody GCTM-2, which binds to human embryonic carcinoma cells and primate ES cells, recognizes an epitope on a keratan sulfate proteoglycan [40,41]. These studies have shown not only that carbohydrates on cell surfaces change with embryological development but also that they can be used to identify different types of stem cells. In addition, there are a number of carbohydrate epitopes that have been found on the mammalian preimplantation embryo. Ofcourse, the tumor rejection antigens and stage specific antigens have been well characterized on human embryos [4], but there are many others such as the TEC antigens (TEC1- TEC4), LeX, LeY, CD46, CD55, and CD59. The TEC antigens have been shown to recognize either the carbohydrate moiety of embryoglycan or a developmentally regulated protein epitope on both mouse and bovine preimplantation embryos [42]. LeX [Galbeta1-4(Fucalpha1-3)GlcNAc] is first detected on the blastomeres of the 8-cell stage of the mouse embryo and LeY [Fucalpha1-2Galbeta1-4(Fucalpha1-3)GlcNAc] is highly expressed on the surface of the mouse blastocyst [43,44]. Also, studies of human preimplantation embryos show that the glycosyl phosphatidylinositol anchored proteins CD46, CD55 and CD59 are expressed on oocytes and plasma membrane [45]. In this study, we analyzed glycans on the cell surface of hESCs using a panel of 14 lectins and SSEA-4 antibody and found that pluripotent hESC surfaces have a large variety of surface carbohydrates that can be targeted for marker sources.

## Conclusion

This work represents a fundamental base to systematically classify pluripotent hESCs, and in future studies these lectins may be used to distinguish differentiated hESC types based on carbohydrate presentation that accompanies differentiation. It will be important to determine the potential of these lectin positive cell types and to identify the cell surface glycoproteins or glycolipids to which these lectins bind. Because there are many different enzymes that add or remove carbohydrate structures on cell surfaces, it will also be interesting to investigate the function of some of these glycosyltransferases in both pluripotent and differentiated hESCs.

## Methods

### Cell culture and passaging

NIH approved hESC lines BG01 and BG02 with normal karyotypes were obtained from BresaGen, Inc. [33]. Cells were grown in DMEM/F12 supplemented with 15% FCS (HyClone), 5% knockout serum replacer, 1× non-essential amino acids, 20 mM L- Glutamine, 0.5 U/ml penicillin, 0.5 U/ml streptomycin, 4 ng/ml FGF-2 (Sigma), (all from Gibco Invitrogen unless otherwise labeled). Human ESCs were maintained on mitotically inactivated primary mouse embryonic fibroblasts (MEF) feeder layers for routine maintenance. Cells were grown in 100 cm tissue culture treated dishes (Falcon). Cells were passaged every 3 days using either a pretreatment with 10 mg/ml collagenase for 2 minutes followed by 0.05% trypsin for 1 minute or manual dissection with a fire pulled Pasteur pipette [33]. Immunocytochemistry was performed on routinely maintained adherent hESC colonies, and flow cytometry was performed using routinely maintained hESC colonies that were first enriched for SSEA-4.

### Enrichment of SSEA-4 positive cells

Cultures of hESCs were grown in 100 cm dishes and trypsin passaged into single cell suspensions as described above. Cells were incubated on ice for 15 minutes in 1:10 dilution of SSEA-4 (MC 813-70, Developmental Studies Hybridoma Bank [DSHA]; Iowa City; in stain buffer (SB). SB consisted of 0.5 ml 0.5 U/ml penicillin, 0.5 U/ml streptomycin, 0.5 ml of 100 mM EDTA, 46.5 ml phosphate buffered saline (PBS) and 2.5 ml fetal bovine serum (FBS). After incubation 10 ml SB was added and cells were resuspended and centrifuged at 3000 × g for 5 minutes. Supernatant was removed, leaving the cell pellet intact, and cells were washed again in SB and resuspended. After centrifugation, a 1:4 dilution of secondary anti-mouse IgG in stain buffer was added to the cell pellet, and the resuspended pellet was incubated on ice for 25 minutes. After incubation 10 ml SB was added to wash the cell pellet, followed by centrifugation (3000 × g) for 5 minutes. This process was repeated two more times in 5 ml SB. Cells were finally resuspended in 500 μl of SB before being applied to a pre-washed magnetic bead column. The flow through from the column was collected and saved for counting, and the retained eluate was collected separately. Both flow through and eluate were brought up to 5 ml in SB after collection and counted [46]. Cells were then subjected to flow cytometry. Flow cytometry results presented here are from hESCs that were used immediately after the enrichment procedure.

### Flow cytometry

Cell surface antigen and carbohydrate expression of SSEA-4+ enriched hESCs was assessed by indirect immunofluorescence detected by flow cytometry to provide a quantitative binding percentage of both SSEA-4 and lectin. Human ESCs were harvested into single cell suspensions using trypsinization as described above. Cells were enriched for SSEA-4 as described above. Then, cells were fixed in 2% paraformaldehyde in 1× PBS. After blocking in 1% bovine serum albumin (BSA) for 30 minutes, cells were placed in sterile conical tubes in aliquots of 500,000 cells each and double stained with one of the 14 lectins at 5 μg/ml and SSEA-4 in a 1:100 dilution. Cells were washed 3 times with PBS and then stained with secondary antibodies that included streptavidin -allophycocyanin (1:250, BD Biosciences; Franklin Lakes, NJ;) for recognition of biotinylated lectins and antigoat Mouse IgG conjugated Alexa 488; (1:2000) for recognition of SSEA-4. These secondary antibodies were chosen so that there would not be overlap in the emission/excitation wavelengths and so that double staining could be performed. Unstained, enriched hESCs and enriched hESCs stained with secondary antibodies alone were used as controls.

Table 1 shows the chosen tested lectins, their commonly abbreviated name, and the specificity of these lectins for their respective monosaccharides to act as a quick reference guide and the concentration of hapten, or competitive sugar, that was used to verify specificity of each lectin for its carbohydrate. Specificity of each lectin has previously been described in detail by Cummings and colleagues [47]. Cytometry was performed using a Beckman Coulter Cytomics FC 500 Flow Cytometer. Data analysis was performed using the RXP Analysis Software by Beckman Coulter and Windows Multi Document Interface for Flow Cytometry (WinMDI 2.8). Three independent assays were carried out using both BG01 and BG02 hESC lines.

### Immunocytochemistry

Immunocytochemistry was used to analyze the localization of cell surface carbohydrate expression and SSEA-4 on routinely maintained adherent cultures of hESCs. Human ESCs were harvested from 100 cm^2 ^dishes by either trypsin/collagenase dissociation or manual dissociation and fixed in 4% paraformaldehyde (Fisher Scientific) in 1× PBS (Gibco) for 30 minutes. After blocking in 1% BSA solution for 30 minutes, cells were equally divided into 4-well chamber slides which had previously been plated with mitotically inactivated MEFs. Cells were then double stained with one of 14 biotinylated lectins (all lectins obtained from Vector Laboratories; Burlingame, CA; 10 μg/ml) and SSEA 4 (MC 813-70, Developmental Studies Hybridoma Bank [DSHA]; Iowa City;1:100) for 20 minutes at 37°C, followed by 3 washes in PBS. Secondary antibodies included streptavidin conjugated Alexafluor 594 (Molecular Probes; Eugene, OR; 1:250 dilution) and antigoat Mouse IgG conjugated Alexa 488 (Molecular Probes; 1:2000 dilution). During staining procedure, cells were kept at 37°C. Cells were washed three times after secondary antibody incubation for 5 minutes in PBS. Cells were then post-stained with 5 ng/ml DAPI (Sigma) to detect cell nuclei and washed overnight in PBS. Controls of unstained cells were obtained by incubation with secondary antibodies alone. All slides were mounted and visualized using a Nikon TS100 inverted microscope. Individual color channels were captured separately and merged in Adobe Photoshop.

### Abbreviations used

human embryonic stem cells (hESCs)

stage specific embryonic antigen-4 (SSEA-4)

inner cell mass (ICM)

N-acetylgalactosamine (GalNAc)

Fibroblast growth factor-2 (FGF-2)

mouse embryonic fibroblasts (MEF)

stain buffer (SB)

phosphate buffered saline (PBS)

fetal bovine serum (FBS)

bovine serum albumin (BSA)

*Concanavalin A *(Con A)

*Phaseolus vulgaris *erythro-agglutinin (PHA-E)

*Phaseolus vulgaris *leuco-agglutinin (PHA-L)

*Sambucus nigra *agglutinin (SNA)

*Arachis hypogea *peanut (PNA)

*Vicia villosa *agglutinin (VVA)

*Maackia amurensis *(MAA)

*Ricinus communis *agglutinin (RCA)

*Wisteria floribunda *agglutinin (WFA)

*Ulex europaeus *agglutinin (UEA)

*Lotus tetragonolobus *lectin (LTL)

*Dolichos biflorus *agglutinin (DBA)

*Hippeastrum hybrid *lectin (HHL)

*Lycopersicon esculetum *tomato (TL)

## Authors' contributions

AV carried out the maintenance and culturing of the hESCs, flow cytometry and immunocyotochemical studies and drafted the manuscript. MM helped in maintenance of hESCs, immunocytochemistry, and aided in manuscript review. KJ participated in flow cytometry set up. SS, IL, and MP participated in the design of the study. SS participated in its design and coordination and helped to draft the manuscript. All authors read and approved the final manuscript.

## Links

Developmental Studies Hybridoma Bank



BD Biosciences



Vector Laboratories



Molecular Probes



## Supplementary Material

Additional File 1 **Histogram plots of other lectins tested. **Figure S1 shows peak shifts of RCA (A), PNA (B), ConA (C), WFA (D), SNA (E), and PHA-L (F) binding in black tracing, and unstained cells in grey fill. Click here for file

Additional File 2 **Flow cytometry plots of other lectins tested. **Figure S2 shows plots of some lectins that have 2 populations of cells such as PHA-L (A), UEA (B), and VVA (C). The plot of CON A (D) indicates only one population of cells showing a positive shift for lectin and SSEA-4 antibody binding, while the plot of DBA (E) and LTL (F) also show only one population of cells with no shift in lectin binding, but a positive shift in the SSEA-4 detection channel. Click here for file
