# Supplementary figures and images for: Lectin binding profiles of SSEA-4 enriched, pluripotent human embryonic stem cell surfaces
Source: BMC Dev Biol. 2005 Jul 21;5:15. doi: 10.1186/1471-213X-5-15 (PMC1182361; doi:10.1186/1471-213X-5-15)

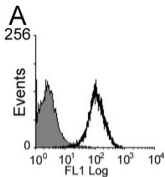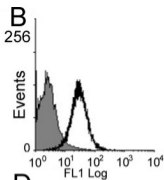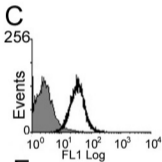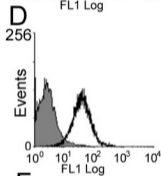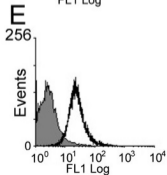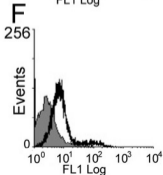

Supplement: Additional File 1 — Histogram plots of other lectins tested. Figure S1 shows peak shifts of RCA (A), PNA (B), ConA (C), WFA (D), SNA (E), and PHA-L (F) binding in black tracing, and unstained cells in grey fill. [file 1471-213X-5-15-S1.PDF]

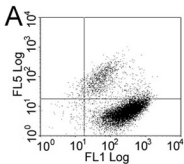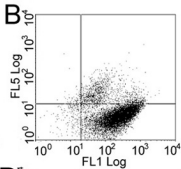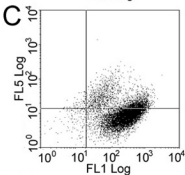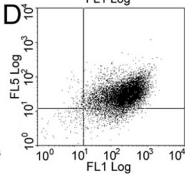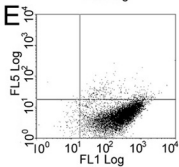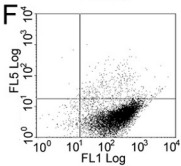

Supplement: Additional File 2 — Flow cytometry plots of other lectins tested. Figure S2 shows plots of some lectins that have 2 populations of cells such as PHA-L (A), UEA (B), and VVA (C). The plot of CON A (D) indicates only one population of cells showing a positive shift for lectin and SSEA-4 antibody binding, while the plot of DBA (E) and LTL (F) also show only one population of cells with no shift in lectin binding, but a positive shift in the SSEA-4 detection channel. [file 1471-213X-5-15-S2.PDF]
